# Spatial Dynamics and Multiscale Regression Modelling of Population Level Indicators for COVID-19 Spread in Malaysia

**DOI:** 10.3390/ijerph19042082

**Published:** 2022-02-13

**Authors:** Kurubaran Ganasegeran, Mohd Fadzly Amar Jamil, Maheshwara Rao Appannan, Alan Swee Hock Ch’ng, Irene Looi, Kalaiarasu M. Peariasamy

**Affiliations:** 1 Clinical Research Center, Seberang Jaya Hospital, Ministry of Health Malaysia, Seberang Perai 13700, Malaysia; fadzly.crc@gmail.com (M.F.A.J.); alanchng1978@gmail.com (A.S.H.C.); irenelooi@yahoo.com (I.L.); 2 Institute for Clinical Research, National Institutes of Health, Ministry of Health Malaysia, Setia Alam 40170, Malaysia; drkalai@moh.gov.my; 3 Crisis Preparedness and Response Center, Disease Control Division, Ministry of Health Malaysia, Putrajaya 62590, Malaysia; mahesh.appannan@gmail.com; 4 Medical Department, Seberang Jaya Hospital, Ministry of Health Malaysia, Seberang Perai 13700, Malaysia

**Keywords:** spatial analysis, regression modelling, COVID-19, Malaysia

## Abstract

As COVID-19 dispersion occurs at different levels of gradients across geographies, the application of spatiotemporal science via computational methods can provide valuable insights to direct available resources and targeted interventions for transmission control. This ecological-correlation study evaluates the spatial dispersion of COVID-19 and its temporal relationships with crucial demographic and socioeconomic determinants in Malaysia, utilizing secondary data sources from public domains. By aggregating 51,476 real-time active COVID-19 case-data between 22 January 2021 and 4 February 2021 to district-level administrative units, the incidence, global and local Moran indexes were calculated. Spatial autoregressive models (SAR) complemented with geographical weighted regression (GWR) analyses were executed to determine potential demographic and socioeconomic indicators for COVID-19 spread in Malaysia. Highest active case counts were based in the Central, Southern and parts of East Malaysia regions of Malaysia. Countrywide global Moran index was 0.431 (*p* = 0.001), indicated a positive spatial autocorrelation of high standards within districts. The local Moran index identified spatial clusters of the main high–high patterns in the Central and Southern regions, and the main low–low clusters in the East Coast and East Malaysia regions. The GWR model, the best fit model, affirmed that COVID-19 spread in Malaysia was likely to be caused by population density (β coefficient weights = 0.269), followed by average household income per capita (β coefficient weights = 0.254) and GINI coefficient (β coefficient weights = 0.207). The current study concluded that the spread of COVID-19 was concentrated mostly in the Central and Southern regions of Malaysia. Population’s average household income per capita, GINI coefficient and population density were important indicators likely to cause the spread amongst communities.

## 1. Introduction

Place, person and time forms the cornerstone of epidemiological investigations towards determining the distribution and determinants of disease occurrence in a particular population [1]. The revolution of infectious-disease epidemiology has enabled outbreak investigators to potentially navigate from using conventional “spot maps” to a more robust computational measurement maneuvered via geographical information systems (GIS), that fundamentally yields “heat maps or choropleths” for visualizing patterns and distribution of disease outbreaks in modern public health practice [2]. Population health data science, real-time data interpretation via GIS and big data applications fundamentally can provide continuous information flow in routine surveillance output for rapid interventions [3,4]. But during pandemic times, as dispersion of infections penetrate even at the smallest level of administrative units within the population, it would be worthwhile to implement mitigation or suppression interventions targeted based on the intensity of the outbreak in a particular place. Having generalized interventions to the whole population would not be cost effective, as governments need to optimize available healthcare supplies and strategies to break the chain of transmissions.

The ongoing coronavirus disease 2019 (COVID-19) has accelerated investments in global health security to mitigate the spread of transmissions at the country and international level. But the biological structure of the virus studied via genomic sequencing have alarmed nations worldwide that the COVID-19 virus will undergo rapid mutations at a pace never seen in history of pandemics, with emerging variants being more likely to cause unprecedented outbreaks in human populations [5,6]. To avoid wide-ranging socioeconomic disruptions, many countries are accelerating vaccination programs for their population, and opted to name COVID-19 as a disease of “endemicity” [7]. As vaccine effectiveness (duration of protection) has yet to be confirmed, individuals will be consistently susceptible to infections. Outbreaks may be precipitated to occur at a logarithmic, exponential or geometrical sequence [8,9]. Under these circumstances, it is crucial to plan and execute targeted interventions by taking into account the space of inhabitants, in the quest to equilibrize between lives and livelihoods.

The bulk of COVID-19 literature to date at the time of writing were mostly prediction and deterministic models of stochasticity related to reproduction numbers, epidemiological features and mortalities [10,11,12,13,14,15]. Although these works are essential to estimate policy and response capacity of health systems during pandemic times, they were not powered to explore the diffusion of the COVID-19 spread according to space [16]. Understanding the aspects of transmissibility based on place would be crucial to execute disease control and harm reduction strategies. While the spatial dispersion of COVID-19 has observed specific flows within regions or territories from different countries [17,18,19,20,21,22,23], their temporal relationships vary, in particular to those related to social determinants of health as a medium of spread for COVID-19 [18,22,24]. Malaysia was nearly successful in containing the outbreak in the previous two waves [25,26], but the current third wave of the pandemic, likely to be caused by spillover effects of a regional state election in late 2020 [27], seemed difficult to contain. To date, there were no spatial epidemiological studies in Malaysia, although incidence cases were heterogenous across different geographies in the country. As spatial dynamics of disease outbreaks were primarily observed within different gradients, determined by location and influenced by local attributes, the current study was aimed to analyze patterns of COVID-19 dispersion in Malaysia and to subsequently determine their relationships with potential demographic and socioeconomic indicators via multiscale autoregressive and geographical weighted regression models.

## 2. Materials and Methods

### 2.1. Study Population, Design and Setting

**Figure 1:**
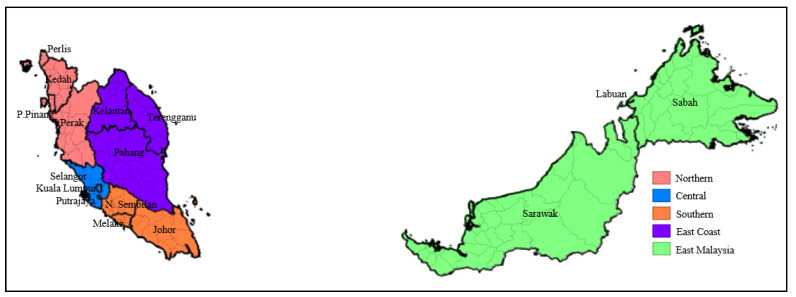
District-wise distribution across states and regions in Malaysia. Dark boundaries represent borders between states; light boundaries represent borders between districts; color shaded areas represent regions.

Between 22 January and 4 February 2021, a nationwide ecological-correlation study was conducted in Malaysia, involving 51,476 active COVID-19 cases spread across 144 districts in 13 states, 3 Federal Territories, and 5 regions (Northern, Central, East Coast, Southern, and East Malaysia) (Figure 1).

### 2.2. Data Source and Indicators

District-wise 14-days moving data of COVID-19 active cases was retrieved from the Ministry of Health Malaysia COVID-19 official webpage (http://covid-19.moh.gov.my, accessed on 4 February 2021); retrieval point—4 February 2021 [28]. The data was obtained during Malaysia’s third wave of the pandemic, just before the re-enactment of a countrywide lockdown. A revised overall forecast of Malaysia’s population stratified by region and district in 2019 was obtained from the Malaysian Population and Housing Census [29]. The total area in square kilometers (km^2^) was obtained from the Malaysian Survey and Mapping Department [30]. Administrative shapefiles and district coordinates were obtained from the Malaysia-Subnational Administrative Districts Data, United Nations Office for Coordination of Humanitarian Affairs [31]. States within Malaysia’s regional boundaries were classified according to the National Population Housing Scheme (PRIMA) [32].

Population density was calculated as the total number of inhabitants in each district per square kilometer [33]. District-level national socioeconomic indicators such as the GINI coefficient and average household income per capita were extracted from the 2019 Household Income and Basic Amenities Survey Report [34]. Ethnic proportions for each district were obtained from the Department of Statistics Malaysia [35]. List of government health clinics that provide coverage to primary healthcare services were retrieved from Malaysia Open Data Portal [36].

### 2.3. Statistical Analysis

**Figure 2:**
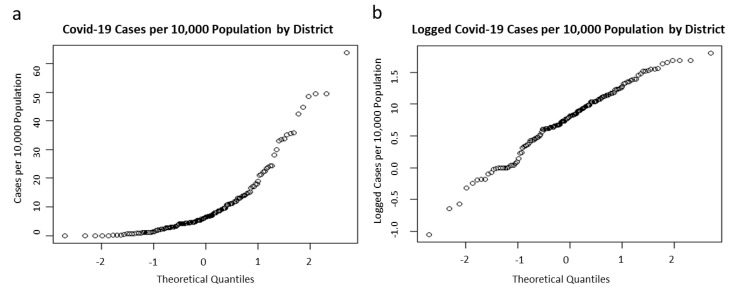
QQ-plots showing (**a**) non-Gaussian distribution of COVID-19 cases per 10,000 population by districts; (**b**) approached normal distribution of logged transformed COVID-19 cases per 10,000 population by districts.

All districts were chosen as the unit of analysis. The total estimated population for all districts in Malaysia as of 2019 was 33,531,200 people [29]. COVID-19 incidence by administrative districts was the main outcome measure in this study. As cases retrieved were count data, the numerical values were subjected to a transformation process in order to facilitate direct applications of spatial analytics method with continuous variables such as Moran’s *I* statistics [37]. Based on this limitation, district-wise incidence of COVID-19 was calculated as the ratio between absolute numbers of cases in each district (numerator) and the total resident population at risk of that particular district according to the 2019 Population and Housing Census of Malaysia (denominator), with the constant set as 10,000 inhabitants. The QQ-plot (Figure 2) showed that the incidence of COVID-19 distribution in this study was not Gaussian, hence a log transformed procedure was applied for the continuous variable to approach a normal distribution. Such techniques were utilized to study spatial structures of COVID-19 in recent investigations [38,39]. Following these requirements, the current study’s main outcome measure was thus the logged transformed COVID-19 incidence; hereinafter, regarded as “COVID-19 incidence” throughout this paper.

At the descriptive level, exploratory spatial analysis was used to generate thematic quintile maps. Next, spatial dependence was tested based on the global Moran index, which identifies spatial autocorrelation that varies between −1 (a negative spatial autocorrelation that identifies occurrence of COVID-19 approaching a scattered pattern) and +1 (a positive spatial autocorrelation that identifies occurrence of COVID-19 approaching a clustered pattern), while a value near zero (0) refers to the occurrence of COVID-19 approaching towards a random distribution (absence of spatial autocorrelation), subjecting that the values were to be statistically significant at *p* < 0.05 [38]. Subsequently, local autocorrelation (local index of spatial association—LISA) was tested using the local Moran index, which verifies the weightage value of the districts with its neighbors through determination of spatial patterns [38]. The generated LISA significance maps would principally identify four quadrants according to the local Moran index values: high–high (districts with high rates of COVID-19 incidence surrounded by neighbors with high rates), low–low (districts with low rates of COVID-19 incidence surrounded by neighbors with low rates), high–low (districts with high rates of COVID-19 incidence surrounded by neighbors of low rates) and low–high (districts with low rates of COVID-19 incidence surrounded by neighbors of high rates), by taking into consideration values of *p* < 0.05 as statistically significant. The high–high and low–low groups were classified as areas of conformity, while high–low and low–high groups indicated epidemiologically transitional areas of COVID-19 incidence [38]. The bivariate Moran’s *I* was determined to examine correlates of COVID-19 incidence.

Multivariate global and local regression models were performed to yield potential indicators of COVID-19 incidence in Malaysia. In view of socioeconomic vulnerabilities, demography and potential barriers to healthcare access during pandemic times, the following indicators were tested as potential predictors for COVID-19 spread in Malaysia: race (percentage of Bumiputera, Chinese and Indians) [35]; GINI coefficient; average household income per capita; coverage of primary healthcare; and population density (logged). The Ordinary Least Square (OLS) model is a global regression model set as a baseline for comparison to other models. OLS excludes weightage of geographical distribution of the pandemic; however, it is capable of determining the relationship between independent variables and COVID-19 incidence counts. To control for spatial effects, additional Spatial Autoregressive (SAR) models were fitted: Spatial Lag Model (SLM) and Spatial Error Model (SEM). Finally, to consider a model that takes into account neighboring cases (local model), a Geographically Weighted Regression (GWR) analysis was conducted. The GWR model has the ability to analyze events based on areal-level variability, hence it is better regarded as a local model. GWR capitalizes the concepts of heterogeneity and nonstationarity over space, hence describes regression coefficients’ variability for each spatial unit over the studied region. For this study, the coefficients synthesized were for each administrative district of Malaysia. The magnitude of the coefficients may actually suggest if an indicator in the model would be protective or being at risk for COVID-19 spread [38]. The diagnostic performance of all models was assessed, and the model that yielded the lowest Akaike Information Criterion (AIC) and the highest R^2^ value was considered as the best fit model [38]. Statistical significance was set at *p* < 0.05. Analyses was conducted using R Studio version 2021.09.2 +382 (R Studio Team, PBC, Boston, MA, USA) [40], Geo Da version 1.18 (Center for Spatial Data Science University of Chicago, IL, USA) [41] and a python implementation of MGWR version 2.2.1 software (ASU School of Geographical Sciences and Urban Planning, AZ, USA) [42].

### 2.4. Conference Presentation

Findings from this study were presented at the 14th National Conference for Clinical Research, 18–20 August 2021, National Institutes of Health, Selangor, Malaysia.

## 3. Results

### 3.1. Spatial Autocorrelation of COVID-19 Incidence

**Figure 3:**
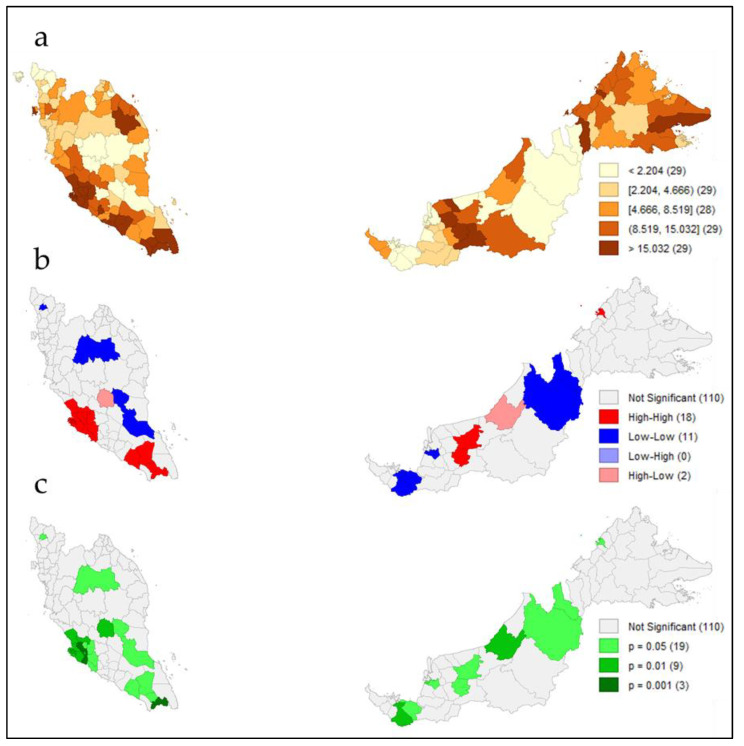
(**a**) Quantile map of spatial distribution of COVID-19 incidence; (**b**) univariate LISA of cluster map; (**c**) univariate LISA significance map of spatial clustering and outliers.

Between 22 January and 4 February 2021, Malaysia reported 51,476 active COVID-19 cases. In an average Malaysian district, there were approximately 10.5 active COVID-19 cases per 10,000 population. The spatial heterogeneity for COVID-19 incidence across 144 districts could be observed from the dual evidenced quintile (Figure 3a) and LISA (Figure 3b,c) maps. The quantile map in Figure 3a showed that approximately 29 districts reporting high rates of COVID-19 incidence (more than 15.03 cases per 10,000 population), with the top 5 districts (mainly concentrated within the states of Selangor, Johor and Sarawak) were Sepang (63.7 cases per 10,000 population), Kulai Jaya (49.48 cases per 10,000 population), Dalat (49.34 cases per 10,000 population), Song (48.55 cases per 10,000 population) and Kanowit (44.67 cases per 10,000 population). Only five districts reported zero COVID-19 incidence cases, all in the state of Sarawak. The highest COVID-19 incidence was mainly concentrated in the Central and Southern regions of Peninsular Malaysia.

In the spatial autocorrelation analysis, the global Moran index was 0.431, with *p* = 0.001, indicating a positive spatial autocorrelation. Subsequently, the local Moran index identified spatial clusters of the main high–high patterns across districts within the state of Selangor and Kuala Lumpur in the Central region, Johor in the Southern region and parts of Sarawak in East Malaysia, while the main low–low clusters were concentrated across rural districts in the East Coast region and the state of Sarawak in East Malaysia (Figure 3b). The LISA enumerates the significance of these clusters (Figure 3c).

### 3.2. Spatial Relationship between National Indicators and COVID-19 Incidence

Table 1 shows the values of Moran’s I results that explored the spatial relationships between probable indicators with COVID-19 incidence for all 144 districts across Malaysia through bivariate LISA analysis. Moran’s *I* was the highest with respect to average household income per capita (Moran’s *I* = 0.46, *p* = 0.001), followed by population density (Moran’s *I* = 0.41, *p* = 0.001), percentage of Indians (Moran’s *I* = 0.36, *p* = 0.001), percentage of Bumiputera (Moran’s *I* = 0.28, *p* = 0.001), percentage of Chinese (Moran’s *I* = 0.20, *p* = 0.001) and GINI coefficient (Moran’s *I* = 0.10, *p* = 0.008).

### 3.3. Multiscale Spatial Regression Models of COVID-19 Incidence

Table 2 exhibits results of Spatial Autoregressive Models (SAR) that include Ordinary Least Squares (OLS), Spatial Lag Model (SLM), Spatial Error Model (SEM) and the Geographically Weighted Regression (GWR) model. The OLS regression analysis yielded three significant indicators for COVID-19 incidence spread in Malaysia, namely the GINI coefficient, average household income per capita and population density. To further consider spatial dependence, complementary spatial models (SLM and SEM) were yielded. Model diagnostics suggested that SEM performed better than priori models with autoregressive coefficient (error lag value λ = 0.46) indicating a significantly higher spatial clustering of COVID-19 occurrence in Malaysia. In addition, the analysis of OLS residual showed spatial dependence with *I* = 0.2615 (*p* < 0.05), thus subjecting hypothesized national indicators to the requirement for an analytical application of a GWR model. The GWR model concluded that COVID-19 incidence spread in Malaysia was highly caused by population density (β coefficient weights = 0.269), followed by average household income per capita (β coefficient weights = 0.254) and GINI coefficient (β coefficient weights = 0.207). The GWR model significantly outweighed the OLS, SLM and SEM models, being the best performing model (R^2^ = 0.661; AIC = 229.435).

**Figure 4:**
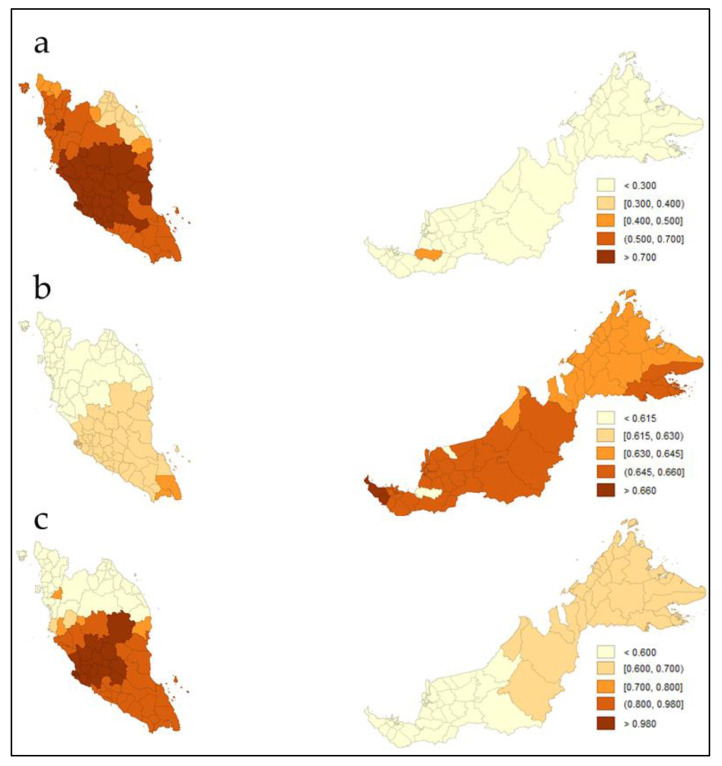
Quantile maps of GWR β coefficients weights (**a**) GINI coefficient; (**b**) average household income per capita; (**c**) population density (logged).

The GWR coefficients quantile maps in Figure 4 have shown similar directions as the relationships found in the global regression models in Table 2. The GINI coefficient, average household income per capita and population density were positively associated with COVID-19 incidence cases. Although population density directed a homogenous pattern with incidence cases being highly concentrated within the Central region of Malaysia, dissimilarities of socioeconomic variables, particularly weightage strengths of GINI coefficient and average household income per capita across neighborhood districts have shown heterogenous patterns correlated with COVID-19 incidence in Malaysia (Figure 4).

## 4. Discussion

The current study was the first in Malaysia to examine the spatial distribution of COVID-19 spread and its relationship with selected population level demographic and socioeconomic indicators of the country. The multiscale regression models yielded three substantial indicators, particularly the GINI coefficient, average household income per capita and population density as probable catalysts that accelerated the unexpected geometrical progression of the third COVID-19 pandemic wave in the country.

Spatial distribution of COVID-19 cases across districts and regions in Malaysia indicated that the spread was not propagating in a uniform pattern. The socioeconomic heterogeneity between districts occupied within regions in Malaysia may have influenced the uneven chaotic spreads, as marked spatial heterogeneity of incidence cases were observed between the East Coast region, East Malaysia (particularly the state of Sarawak) and the Central region of Malaysia, among which the latter represents a highly industrialized, economic and financial epicenter of the country. This finding was consistent with previous studies from Brazil [38], India [43], China [44], Malawi [22] and England [19]. Plausible explanations for such consistencies can be attributed to selective migration of people, facilitating large territorial flows from neighboring regions or states to the Central region of the country to capture potential commercial or labor markets that could have substantially cultivated dense populations in those areas, causing greater human mobility and interactions. Such relocation behaviors on existing geographical networks sustains a super-spreader incubator for pathogenic events within densely contemporary societies [33,45]. With industrialization being highly clustered within the Central region of Malaysia, the implementation of mass testing policy by the government in factories, industries and migrant workers have substantially reported a greater number of cases [46]. Consistent with the impact of urban population density, coupled with economic development and integrated public transportation services that catalyzed higher human mobility, these elements could have escalated opportunities for spatial transmission of COVID-19.

In contrast, the relatively lower incidence of COVID-19 cases across the spatial gradient of rural districts in the East Coast and East Malaysia regions may be explained by lower testing rates as compared to urbanized regions, similarly argued in a previous study [21]. The current study finding was consistent with a previous study from Malawi [22]. Migratory behaviors to urban areas are often observed in younger aged populations in the quest to capture the job market within fast economic growing cities and metropolitans [22], while older populations are more comfortable to reside in their home villages within the rural gradient, mainly inclined with agriculture, fishing or forestry activities. These occupations somewhat sustain lower mobility and interactions amongst people; thus, they have lower probability or reduced transmission rates of infection.

As spatial unevenness or “centralization” of societies are fundamentally dependent on resources for livelihoods (e.g., job opportunities, food, housing) and communities’ social developmental factors (urban or rural setting), their dissimilarities could be temporally attributed to the measures of economic theory, the GINI coefficient [47]. The current study was sufficiently powered to establish the temporal relationship between GINI coefficient and COVID-19 spread across geographically weighted demographic densities of the Malaysian population, consistent with previous studies from Brazil [48,49]. A notable real-life interpretation of the GINI coefficient predominantly lies in the relatively lower metric for Malaysia [29]. As populations’ living circumstances improved with narrowed income inequalities, Malaysians were able to cope with the unexpected pandemic crisis, as resources and social development were developing at an equilibrium across all regions in Malaysia. This may have prompted equal testing opportunities and accessibility to healthcare, thus reporting a higher number of cases. Nevertheless, the caveat of such metrics should be interpreted with caution. Firstly, as the GINI coefficient measures variations between population counts alongside their inhabited areas, they could be intrinsically influenced by a variety of spatial scales or measurement tools used in different studies. Secondly, the temporal variation of population distribution is conceptually measured by the coefficient of variation, which causes disparities of mean population abundance [50]. Such a proviso within population health metrices, although it showed significance in the current study, may fail to establish temporality in future hypothetical associations when other mediators or confounding variables are included in the models. However, they could be descriptively useful to postulate variation patterns for human selective migratory behaviors and their indirect impact on the capability to accentuate transmissions in densely populated areas, as observed in this study.

In the GWR analysis, average household income per capita was associated with COVID-19 incidence in Malaysia. This finding was consistent with a previous study from Brazil [38]. Regions with higher population income would ideally exhibit greater incidence cases as these regions would have better networks of health services, facilitating people’s easy access to diagnostic testing for COVID-19. There was no significant relationship between COVID-19 incidence and coverage to primary healthcare services, contradicting the results observed in a previous study [38]. With Malaysia’s growing household income per capita and narrowed GINI coefficient [29], it was possible to observe fair access to health services to the whole population in terms of quality and quantity. While the urban public could opt for health services from both subsidized government or private clinics with a fee for health services and testing, in the rural districts of Malaysia the highly subsidized healthcare services by the government [51] ensured the availability of free diagnostic testing for COVID-19. With such affordability and access to health services, the non-significance of healthcare coverage to influence incidence cases could be highly anticipated in the regression model, as observed in the current study. In addition, rigorous contact tracing activities executed by public health Malaysia provided early identification of COVID-19 cases within communities, thus making the accessibility or coverage to primary healthcare facilities by local inhabitants for COVID-19 testing to have a “negligible effect,” as observed in this study.

Studies show racial or ethnic differences are important demographic indicators for disease transmissions, as different races represent different cultures or behaviors that could affect contact rates, transmissions rates or perceptions to be vaccinated [52,53,54]. While the current study indicated transmission rates amongst ethnic Indians to be relatively high, compared to Bumiputera and ethnic Chinese, these associations were suppressed to a negligible effect at the local spatial level via the regression models and GWR-weighted analysis. Plausible explanations on higher susceptibility by ethnic Indians to disease transmissions may be attributed to the fact that minority populations would have higher proportions of people with lower socioeconomic status, thus escalating their risks for susceptibility to infections [54]. In contrast, Bumiputera and ethnic Chinese differ in social patterns, mixing within the same communities and have greater mobility during the festive and holiday seasons. However, these attributes did not show any statistical significance in the OLS, SLM and SER models. The coefficient weights were negative, suggesting that race attributes had a low effect on transmission patterns at the spatial-local level. The plausibility of these situations may have been mediated by socioeconomic variables in the model (as justified earlier) and community mitigation measures that were executed simultaneously for the whole population and fairly by the government of Malaysia during the COVID-19 pandemic. But this finding was in contrast with investigations across different ethnic origins from other countries such as Hispanics, Caucasians or African-Americans, where transmissibility rate of infections was high and strongly influenced by the race or ethnicity attribute as compared to Asians [54]. In this circumstance, lower susceptibility of Asians to infections was shown to be attributed to biological factors that seemed to exhibit protective effects [52,54].

This study facilitated an analysis using smaller administrative units (districts in Malaysia), thus enabling more detailed clusters to be synthesized for interpretation. The adoption of spatial autoregressions, in addition to GWR analysis has made it possible to determine crucial population-level socioeconomic indicators that serve as catalysts for the spread of COVID-19 in Malaysia. However, the limitations of this study should be acknowledged. First, the study duration was relatively short, thus temporal patterns of COVID-19 incidence across geographies could not be explored to examine a more chronological sequence of the infection spread, yet to determine other probable mediating attributes such as temperature or climatic factors, interventions over time and vaccination programs to influence the COVID-19 spread. Second, shortcomings of data accessibility and availability for more comprehensive analyses should be acknowledged. Data at different spatial scales such as at states or regional levels that could not be fitted to a more local level, such as at the district level, may have missed opportunity for exploring secondary, mediating or confounding effects with other demographic or socioeconomic variables. These limitations are a common pitfall for spatial analytical studies that utilize secondary data sources. Table 3 provides a list of possible covariates that may pose secondary, mediating or confounding effects to the current study findings, and it is recommended that these attributes be explored in future investigations. Third, the ecological-correlation study design that used secondary data sources could not establish causality, and only explored relationships at the population aggregate level, not at the individual-level. Fourth, the tested variables which include selected population demographics, socio-economic characteristics and population density differ between countries, hence anticipating consistencies between regions would not be possible, even if similar methodologies or variables are replicated and tested in future studies from different countries. However, such tested variations could be evidenced as a country-specific case study that would be crucial to implement targeted approach for control measures based on population’s specific attributes and behaviors.

## 5. Conclusions

In conclusion, the findings of this study provide an understanding of the geospatial characteristics and distributions of the COVID-19 spread in Malaysia. The rigorous spread of COVID-19 was mainly found in the Central, Southern and part of East Malaysia regions in the country, most with an urbanized geography of high population density and average income per capita. This finding could be used to plan appropriate tailored interventions for transmission control between regions or districts in Malaysia by optimizing sufficient resources; with territorial-based mitigation or suppression strategies to flatten the epidemic curve by taking into account the effects of populations’ social determinants of health.

## Figures and Tables

**Table 1:** Bivariate Moran’s *I* of COVID-19 Incidence According to National Indicators.

Indicators	Moran’s *I* Value (*p*-Value)
GINI coefficient	0.10 (0.008)
Average household income per capita	0.46 (0.001)
Coverage to primary healthcare	0.01 (0.396)
Percentage of Bumiputera	0.28 (0.001)
Percentage of Chinese	0.20 (0.001)
Percentage of Indian	0.36 (0.001)
Population density (Logged)	0.41 (0.001)

**Table 2:** Key Regression Indicators of COVID-19 Incidence in Malaysia.

Indicators	OLS Model			SLM Model			SEM Model			GWR Model
	*β*	SE	*p*-Value	*β*	SE	*p*-Value	*β*	SE	*p*-Value	*β* (Mean)
GINI coefficient	2.261	0.903	0.013	1.931	0.837	0.021	1.558	0.833	0.041	0.207
Average household income per capita	0.271	0.073	<0.001	0.266	0.067	<0.001	0.263	0.066	0.003	0.254
Coverage to primary healthcare	0.017	0.014	0.231	0.015	0.013	0.257	0.019	0.012	0.111	0.007
Percentage of Bumiputera	−0.064	0.035	0.066	−0.055	0.032	0.087	−0.052	0.035	0.136	−1.526
Percentage of Chinese	−0.060	0.034	0.083	−0.049	0.032	0.124	−0.047	0.035	0.183	−1.059
Percentage of Indian	−0.052	0.035	0.137	−0.053	0.032	0.104	0.043	0.036	0.227	−0.130
Population density (Logged)	0.388	0.097	<0.001	0.340	0.092	<0.001	0.450	0.120	<0.001	0.269
Model Performance										
Number of observations	144			144			144			144
Log likelihood	−117.936			−112.277			−108.317			−105.645
Akaike Information Criterion (AIC)	251.871			242.553			232.635			229.435
R square	0.552			0.593			0.630			0.661
Lag Coefficient (ρ)	-			0.264			-			-
Error Lag Value (λ)	-			-			0.460			-
Jarque–Bera	12.584 (*p* = 0.002)			-			-			-
Breusch–Pagan	15.805 (*p* = 0.027)			15.832 (*p* = 0.026)			15.868 (*p* = 0.026)			-
Koenker–Bassett	13.446 (*p* = 0.006)			-			-			-

OLS—Ordinary Least Squares; SLM—Spatial Lag Model; SEM—Spatial Error Model (SEM); GWR—Geographically Weighted Regression.

**Table 3:** Potential Indicators for Future Exploration.

Potential Indicators
1. Unemployment 2. Proportion of population with secondary education or less 3. Territorial (area-based) occupations 4. Proportion of older aged persons (more than 60 years old) 5. Proportion of median household income (B40 low-income group) 6. Proportion of median household income (M40 middle-income group) 7. Proportion of median household income (T20 high-income group) 8. Frequency of contacts 9. Human mobility intra-districts 10. Human mobility inter-districts 11. Proportion of urban population 12. Proportion of rural population 13. Urbanization growth rate 14. Vaccination coverage 15. Climatic factors

Note: B40—Bottom 40% household income group (household income range < MYR 4850 per month); M40—Middle 40% household income group (household income range MYR 4850–10,959); T20—Top 20% household income group (household income range ≥ MYR 10960) [34].

## Data Availability

Publicly available datasets were utilized in this study. These data can be found here: https://covid-19.moh.gov.my/ (accessed on 4 February 2021); https://www.dosm.gov.my/v1/ (accessed on 4 February 2021); https://www.jupem.gov.my/ (accessed on 4 February 2021).
